# Oral supplementation of *Pediococcus pentosaceus* SMM847 ameliorates hyperuricemia in rats and alcohol-induced injury in mice

**DOI:** 10.1128/aem.00246-26

**Published:** 2026-03-18

**Authors:** Yuxin Cao, Linfeng Wang, Cailing Ren, Chenxi Li, Yang Liu, Tong Zhang, Mengjiao Luo, Pan Zhao, Ruijuan Li, Jun Fu, Jia Yin

**Affiliations:** 1 Hunan Provincial Key Laboratory of Animal Intestinal Function and Regulation, Hunan International Joint Laboratory of Animal Intestinal Ecology and Health, College of Life Sciences, Hunan Normal University 554899, Changsha, China; 2 State Key Laboratory of Microbial Technology, Shandong University 520252, Qingdao, China; Universita degli Studi di Napoli Federico II, Portici, Italy

**Keywords:** *Pediococcus pentosaceus*, hyperuricemia, acute alcohol intoxication, probiotics

## Abstract

Excessive dietary purine intake can lead to hyperuricemia, facilitating the precipitation and deposition of monosodium urate crystals in joints, which subsequently triggers inflammatory cascades leading to gout. Alcohol consumption exacerbates this condition by accelerating purine metabolism and impairing renal uric acid excretion, thereby further increasing serum uric acid concentrations and aggravating gout severity. In this study, *Pediococcus pentosaceus* SMM847 was screened based on its inosine degradation efficiency. Notably, the strain SMM847 also exhibited alcohol dehydrogenase and aldehyde dehydrogenase activities, as well as a substantial capability for ethanol degradation. In a hyperuricemic rat model, administration of SMM847 significantly reduced serum uric acid levels. Furthermore, in an ethanol-stressed mouse model, SMM847 markedly decreased blood ethanol concentrations, restored hepatic metabolic function, and enhanced endogenous antioxidant capacity, thereby counteracting ethanol-induced disturbances in nutrient absorption and systemic metabolism. Importantly, SMM847 showed no hepatotoxicity and effectively mitigated alcohol-induced histopathological liver injury. By concurrently targeting hyperuricemia and acute alcohol intoxication, this study identifies SMM847 as a promising probiotic candidate for the management of gout and alcohol-related metabolic disorders.

**IMPORTANCE:**

This study presents the probiotic strain *Pediococcus pentosaceus* SMM847 as a promising therapeutic agent for two clinically significant conditions: hyperuricemia and acute alcohol intoxication. The strain mediates uric acid reduction through modulation of purine catabolism pathways. It is particularly noteworthy that SMM847 exhibits ethanol-catabolizing activity, attenuates alcohol-elicited systemic metabolic dysregulation, and exerts hepatoprotection without intrinsic toxicity. The ability of this single bacterial entity to simultaneously target dual pathological pathways—uric acid metabolism and alcohol-induced metabolic disruption—offers a novel and translatable approach for the development of functional foods or pharmaceutical interventions aimed at promoting integrated metabolic and hepatic health.

## INTRODUCTION

With economic development and rising dietary standards, foods rich in purines, such as meat and seafood, have become regular components of the daily diet. In the human body, purines are metabolized into uric acid, and elevated blood uric acid levels significantly increase the risk of gout (1). To date, the global prevalence of gout and hyperuricemia continues to rise annually (2). Compared to preventive dietary restrictions, pharmacological interventions offer better patient compliance. Moreover, combining medication with dietary control yields more significant clinical outcomes.

Dietary purine intake contributes to approximately one-third of daily urate production (3). Uric acid, the final metabolite of purine metabolism, is predominantly generated through the oxidation of xanthine by xanthine oxidase. Xanthine itself is produced via the oxidation of inosine and guanine, catalyzed by xanthine oxidase and guanosine monophosphate dehydrogenase, respectively (4). Disruptions in purine metabolism or impaired renal excretion can lead to elevated serum uric acid levels. Persistent hyperuricemia can result in the deposition of monosodium urate crystals in joints and soft tissues, thereby triggering acute gout attacks (5). Recent studies have indicated that gout has a genetic basis, where genetic characteristics can influence both the excretion of uric acid and the immune response to uric acid in the joints (6). Current therapeutic agents are broadly categorized into anti-inflammatory agents (e.g., NSAIDs and colchicine) and urate-lowering drugs (e.g., xanthine oxidase inhibitors like allopurinol) (7). Medications such as allopurinol can effectively control uric acid levels with few side effects. If needed, anti-inflammatory medications can be used to treat acute attacks. Meanwhile, alcohol consumption exacerbates gout risk through multiple mechanisms: ethanol accelerates purine synthesis (8) and metabolic turnover, stimulates lactate production—which competitively inhibits renal uric acid excretion—and introduces additional purines formed during fermentation (9).

To date, numerous studies have explored disease management using bacteria, establishing well-defined oral administration protocols that support the use of probiotics in disease treatment and prevention (10). For instance, we previously utilized *Pediococcus pentosaceus* SMM914, a probiotic bacterium originally isolated from sow milk, based on its antioxidant activity and demonstrated potential to modulate chronic obstructive pulmonary disease progression (11). This strain has also been shown to alleviate weaning stress in piglets (12). The hepatoprotective effect of *Pediococcus pentosaceus* CGMCC 7049 against ethanol-induced liver injury has been reported. This protection is attributed to its ability to restore gut microbiota homeostasis, modulate short-chain fatty acid metabolism, and strengthen the intestinal barrier—mechanisms involving the reduction of systemic inflammatory responses (13). Extensive research demonstrates that probiotics like *Lacticaseibacillus paracasei* ameliorate hyperuricemia by degrading uric acid and modulating gut microbiota, short-chain fatty acid production, and uric acid transporter expression (14, 15).

In contrast to existing approaches that typically focus on post-consumption metabolic regulation or target only a single pathological pathway, this study adopts a dual-pronged preventive strategy. By utilizing gut microbes to degrade purines and alcohol prior to systemic absorption, our approach mitigates the rise in serum uric acid and minimizes the side effects commonly associated with conventional drugs.

## RESULTS

### *In vitro* assessment of microbial strains with potential uric acid-reducing and alcohol-metabolizing capacities

**Fig 1:**
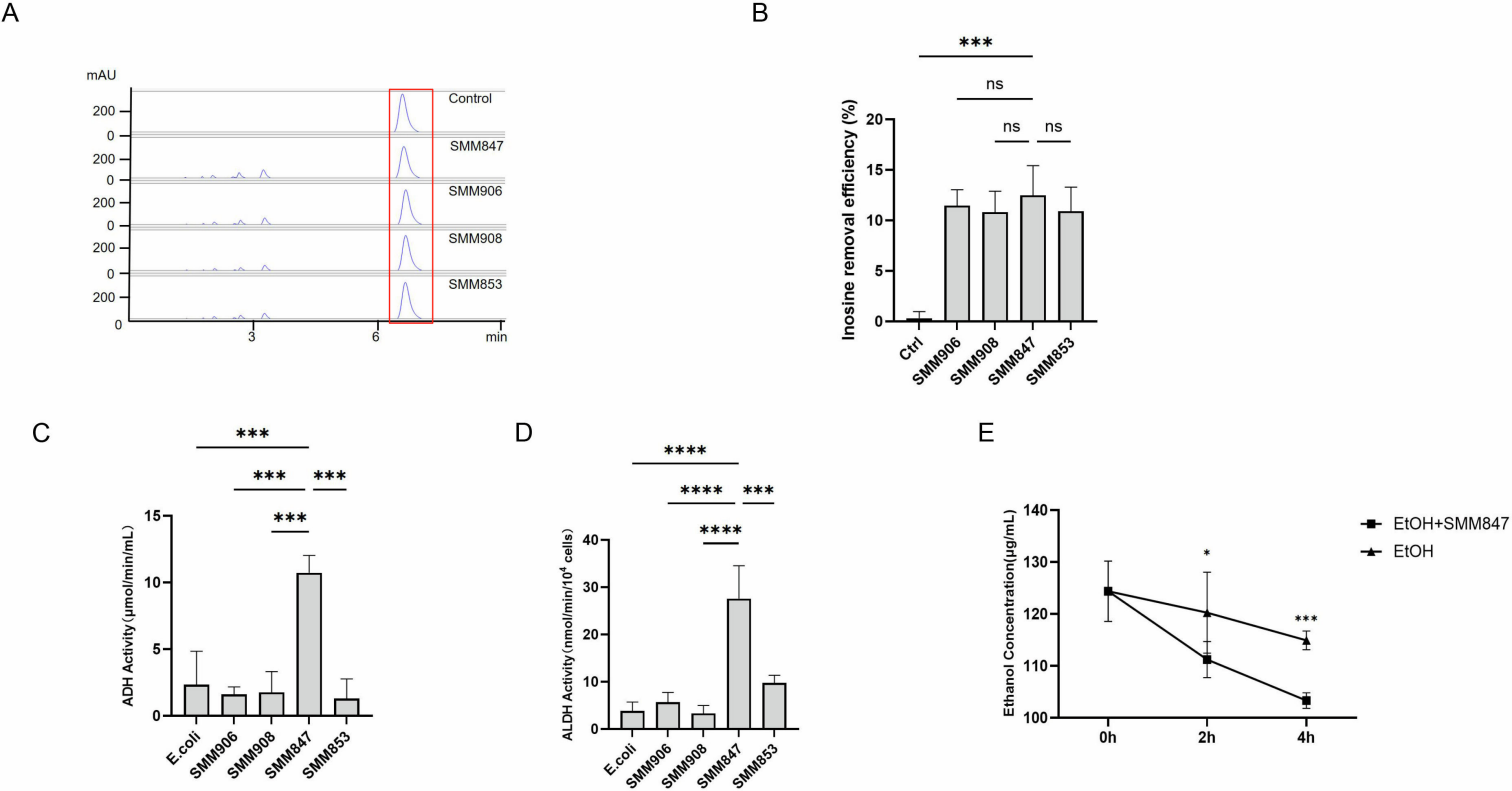
*In vitro* assessment of microbial strains with potential alcohol-metabolizing and uric acid-reducing capacities. (**A**) HPLC profiles of inosine removal by the samples following strain incubation, the control is incubation without strain. (**B**) The removal efficiency histogram of inosine from the samples shown in panel **A**. (**C**) Enzyme activity assay of alcohol dehydrogenase (ADH) in the five bacterial strains. (**D**) Enzyme activity assay of aldehyde dehydrogenase (ALDH) in the five bacterial strains. (**E**) Time-resolved degradation profile of ethanol *in vitro* comparing SMM847 versus sterile medium control, depicted as residual ethanol concentration versus incubation time. Results are presented as mean values ± SD. *N* = 3, statistical significance was determined by *t*-test. *P < 0.05, **P < 0.01, ***P < 0.001, and ****P < 0.0001.

Gout is primarily caused by hyperuricemia, which leads to monosodium urate crystallization and acute inflammation. As inosine is one of the key precursor substances in uric acid production, we screened bacterial strains based on their inosine degradation efficiency to identify candidates with potential uric acid-reducing potential. A total of 80 *Pediococcus pentosaceus* strains from our sow milk-derived collection were evaluated (12). Using high-performance liquid chromatography (HPLC), we quantified inosine levels by measuring the absorption peak area at the retention time corresponding to the standard inosine samples before and after bacterial treatment (Fig. 1A). Based on the reduction in peak areas, the strain SMM847 was identified as exhibiting the highest inosine degradation efficiency (Fig. 1B).

Since alcohol consumption is a major factor for hyperuricemia due to its inhibition of uric acid excretion, we further assessed the alcohol-degrading capacity of SMM847 and four other strains. Given the central role of alcohol dehydrogenase (ADH) and aldehyde dehydrogenase (ALDH) in alcohol metabolism, we first measured their enzymatic activities of ADH and ALDH in the five bacterial strains (Fig. 1C and Fig. 1D). The results showed that SMM847 possesses substantial basal activities of both ADH and ALDH. Subsequent *in vitro* alcohol degradation assays further confirmed its ability to significantly reduce alcohol levels (Fig. 1E). In summary, these preliminary findings identify SMM847 as a promising probiotic strain with dual uric acid-reducing and alcohol degradation functions, providing a foundation for subsequent *in vivo* experiments.

### The capability of SMM847 to traverse the gastrointestinal barrier is confirmed in mice model

**Fig 2:**
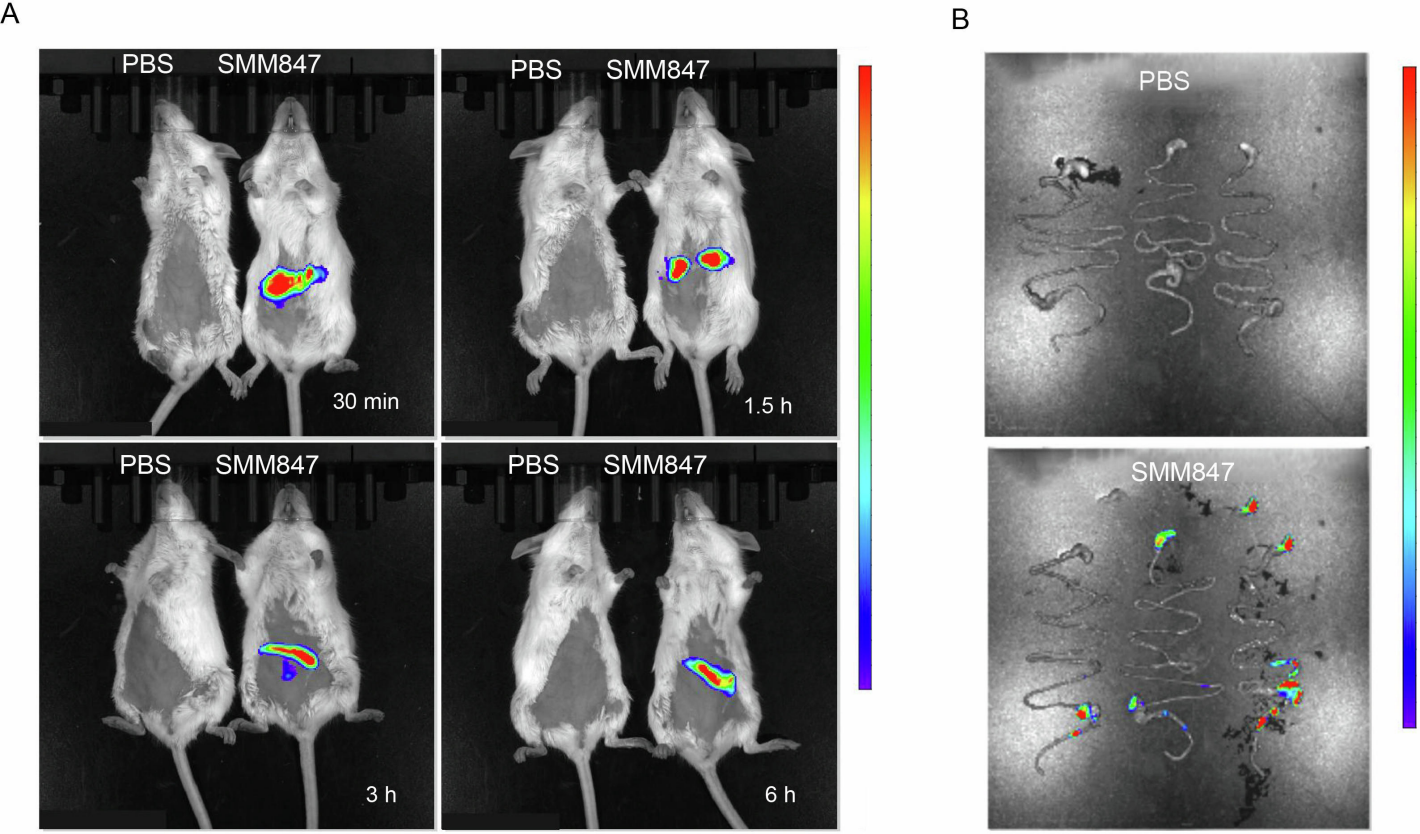
Live imaging of mice after orally administrating SMM847 at 30 min, 1.5 h, 3 h, and 6 h. (**A**) Comparative *in vivo* imaging of mice following oral gavage of CY5.5-d-lys-SMM847 versus the phosphate-buffered saline (PBS) control group (exposure time: 0.5; binning factor: 8; excitation filter: 675; emission filter: 720; f number: 2; field of view: 13.4). (**B**) Comparative *ex vivo* intestinal imaging at 6 h post-oral gavage of CY5.5-d-lys-SMM847 versus the PBS control group (exposure time: 0.5; binning factor: 8; excitation filter: 675; emission filter: 720; f number: 2; field of view: 22.9).

The effective duration and colonization capacity of SMM847 in mice were investigated using *in vivo* imaging to track its intestinal distribution. Following oral gavage of Cy5.5-d-lys-labeled SMM847, real-time fluorescence imaging under anesthesia showed a significant signal in the stomach of nude mice compared to controls. To accurately assess gastrointestinal distribution, *ex vivo* imaging was conducted (Fig. 2A). The results showed that the fluorescent signal was primarily located in the ileum at the 6-h time point, demonstrating that SMM847 possesses the ability to traverse the gastric acid barrier and persist in the intestinal tract (Fig. 2B).

### SMM847 significantly reduces serum uric acid levels in hyperuricemic rat model

**Fig 3:**
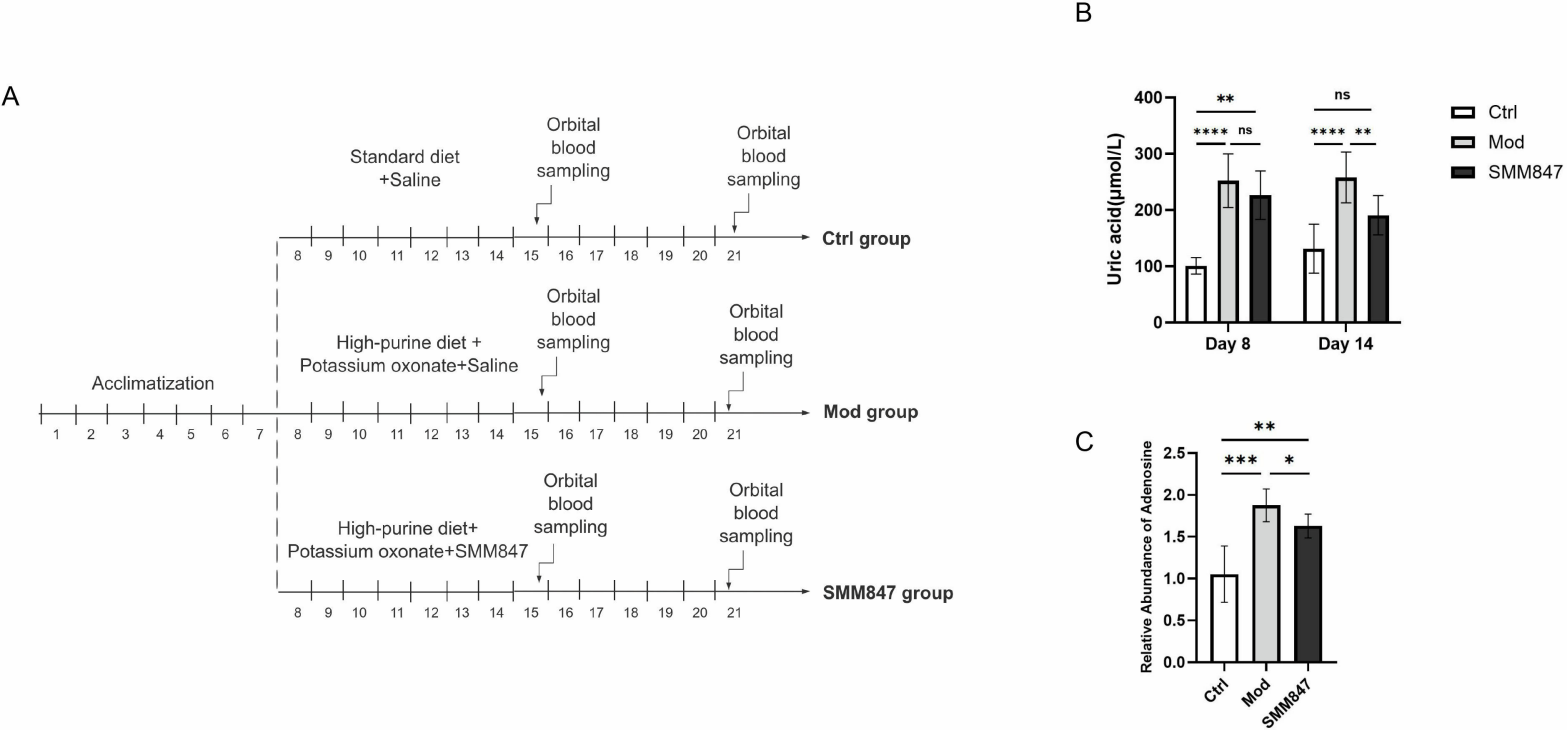
The effect of SMM847 on uric acid levels in rats with hyperuricemia-induced models. (**A**) Experimental design and serum uric acid measurement in a hyperuricemic rat model. Following a 1-week acclimatization, rats were subjected to a 2-week modeling period. The SMM847 group received a normal diet, potassium oxonate, and 1 × 10^9^ CFU SMM847 bacterial suspension. The model group received a high-purine diet, potassium oxonate, and saline. The control group received a standard diet and saline. Serum uric acid levels were measured via orbital blood sampling at endpoint. (**B**) Determination of uric acid content in the control group, model group, and SMM847 group. (**C**) Relative abundance of adenosine in rat intestinal contents. Twenty-four male Sprague-Dawley rats (8–10 weeks old) were randomly allocated into three groups (*N* = 8/group) following a 1-week acclimation. All animals were housed under specific pathogen-free conditions. Throughout the 2-week modeling period, the groups received distinct interventions: the SMM847 group received a high-purine diet, potassium oxonate injections, and oral gavage of SMM847 suspension (1 × 10^9^ CFU); the model group received the high-purine diet, potassium oxonate injections, and saline gavage; the control group received a standard diet, vehicle injections, and saline gavage. Results are presented as mean values ± SD. *N* = 8, statistical significance was determined by Tukey’s multiple comparison test. *P < 0.05, **P < 0.01, ***P < 0.001, and ****P < 0.0001.

To investigate the uric acid-lowering potential of SMM847 *in vivo*, we established a hyperuricemia rat model by administering a customized high-purine diet along with potassium oxonate, which acts as a urate oxidase inhibitor to block uric acid metabolism, thereby ensuring a reliable induction model (Fig. 3A). Model validation was confirmed on both day 8 and day 14, as serum uric acid levels in the model group were significantly elevated compared to the control group. After the hyperuricemic state was stably established, intervention with SMM847 led to a significant reduction in serum uric acid levels relative to the model group (Fig. 3B). As shown in the figure, on day 8, a statistically significant difference was observed between the SMM847 and control groups, whereas no such difference was found between the SMM847 and model groups. However, by day 14, a marked statistical difference emerged between the SMM847 and model groups, while the SMM847 group was no longer significantly different from the control group. Metabolomic analysis revealed that the high-purine diet successfully induced a significant accumulation of adenosine, a key intermediate in purine metabolism, in rats, confirming the presence of a purine metabolic burden in the model group. However, concurrent supplementation with SMM847 in the diet alleviated this trend, leading to a reduction in adenosine levels (Fig. 3C). This result directly demonstrates that SMM847 can effectively mitigate the purine metabolic stress induced by a high-purine diet. These *in vivo* findings demonstrated that SMM847 administration effectively lowers circulating uric acid levels under hyperuricemic conditions, highlighting its potential as a probiotic for mitigating hyperuricemia.

### Effects of SMM847 in alcohol-exposed mice model

**Fig 4:**
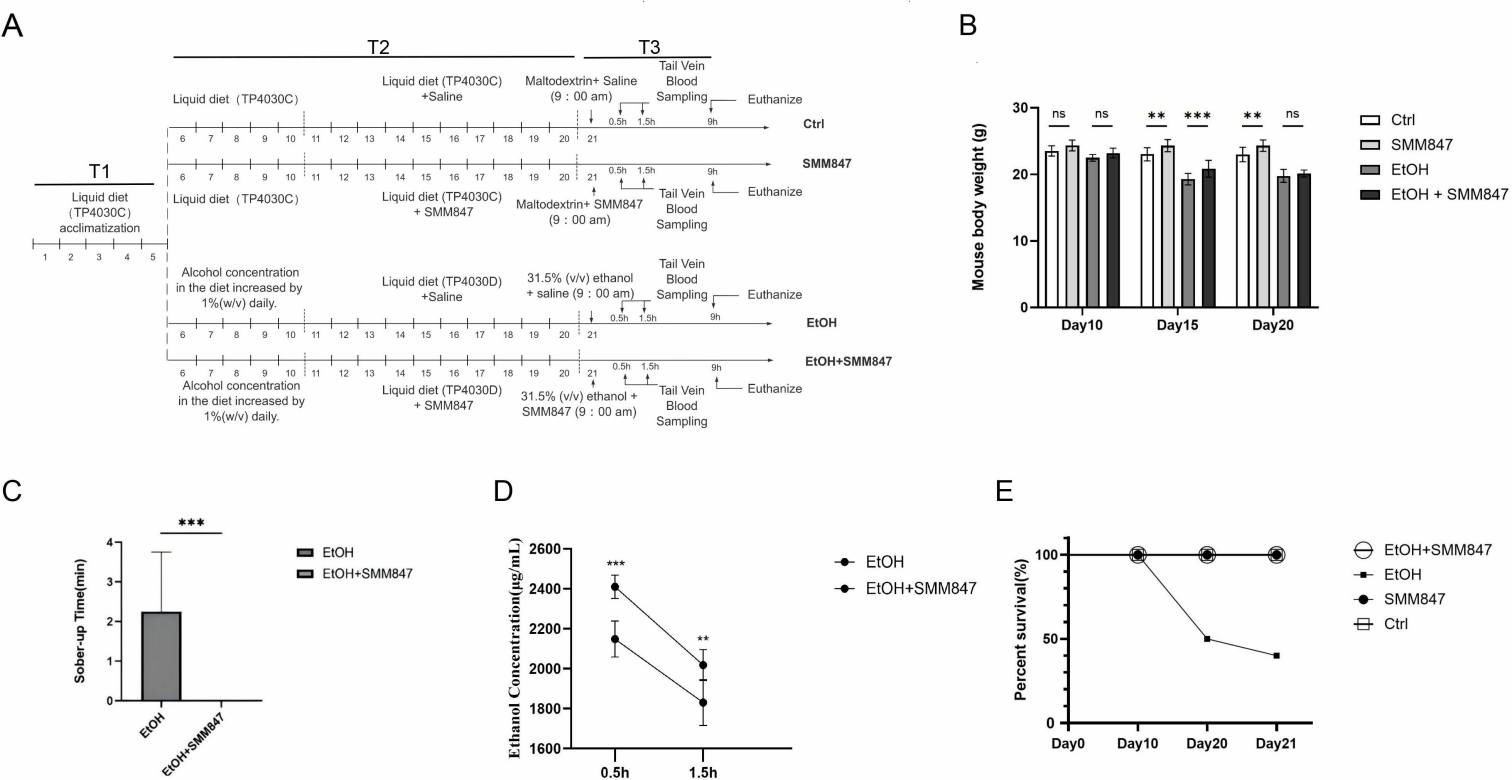
The effect of SMM847 on the alcohol-modeled mice. (**A**) Experimental timeline and treatment protocol for the chronic-binge ethanol feeding model with SMM847 intervention. Following a 5-day adaptation period with Lieber-DeCarli control liquid diet (TP4030C), mice were randomly divided into four groups (*N* = 10). From days 6 to 10, the Ctrl and SMM847 groups remained on the control diet, while the EtOH and EtOH + SMM847 groups underwent a 5-day ethanol adaptation (increasing by 1% per day to 5% wt/vol). From days 11 to 20, the Ctrl and SMM847 groups received the control diet (TP4030C), whereas the EtOH and EtOH + SMM847 groups received the ethanol liquid diet (TP4030D). All groups received corresponding daily gavages (saline or 1 × 10^9^ CFU SMM847). On day 21, groups received either maltodextrin (9 mg/kg) or acute ethanol gavage (5 g/kg, 31.5% vol/vol), followed by respective bacterial or saline gavages. Tail vein blood was collected at 0.5 h and 1.5 h post-gavage into heparinized tubes and stored at 4°C. Mice were euthanized at 9 h after the final gavage for serum and liver collection. (**B**) Analysis of weight changes in mice under four different conditions. (**C**) Comparison of decanter time in drunken mice with and without SMM847. (**D**) Changes of blood ethanol over time in mice with and without SMM847. (**E**) Comparison of survival rates in drunken mice. Results are presented as mean values ± SD. *N* = 10, statistical significance for panels **B**, **C**, and **E** was determined by Tukey’s multiple comparison test. Statistical significance for panel **D** was determined by Šídák multiple comparison test. *P < 0.05, **P < 0.01, ***P < 0.001, and ****P < 0.0001.

To investigate the protective effects of SMM847 on acute alcohol consumption in mice, we established an alcohol-exposed mice model (Fig. 4A). During the alcohol modeling period, body weight was monitored across all groups. While the EtOH group showed significantly lower body weight than the EtOH + SMM847 group at the mid-point of modeling (Fig. 4B), this difference was no longer statistically significant by the end of the modeling period. In the acute alcohol challenge, SMM847 supplementation prevented the complete loss of righting reflex (LORR) (Table 1) and significantly shortened the duration of intoxication (Fig. 4C). Blood ethanol analysis conducted on the final day revealed that SMM847 significantly reduced ethanol concentrations at both 0.5 and 1.5 h after acute alcohol gavage (Fig. 4D). Survival analysis exhibited a notable decline in the EtOH group, whereas all SMM847-supplemented groups maintained a 100% survival rate (Fig. 4E). These findings suggest that SMM847 may facilitate intestinal ethanol metabolism, thereby mitigating alcohol-induced toxicity and improving survival outcomes.

**TABLE 1:** SMM847 pretreatment prevents alcohol-induced loss of righting reflex (LORR)

Group	Initial number^*b*^	Animals subjected to gavage^*c*^	Incidence of LORR	Mortality rate post-gavage
EtOH	10	5	2/5 (40%)	2/5 (40%)
EtOH + SMM847^*a*^	10	10	0/10 (0%)	0/10 (0%)

^*a*^ *P* < 0.05 versus EtOH group (Fisher's exact test).

^*b*^ Number of animals at the start of the experiment.

^*c*^ Number of animals that actually received the ethanol gavage.

### SMM847 mitigates alcohol-induced pathological damage to liver tissue and exerts hepatoprotective effects

**Fig 5:**
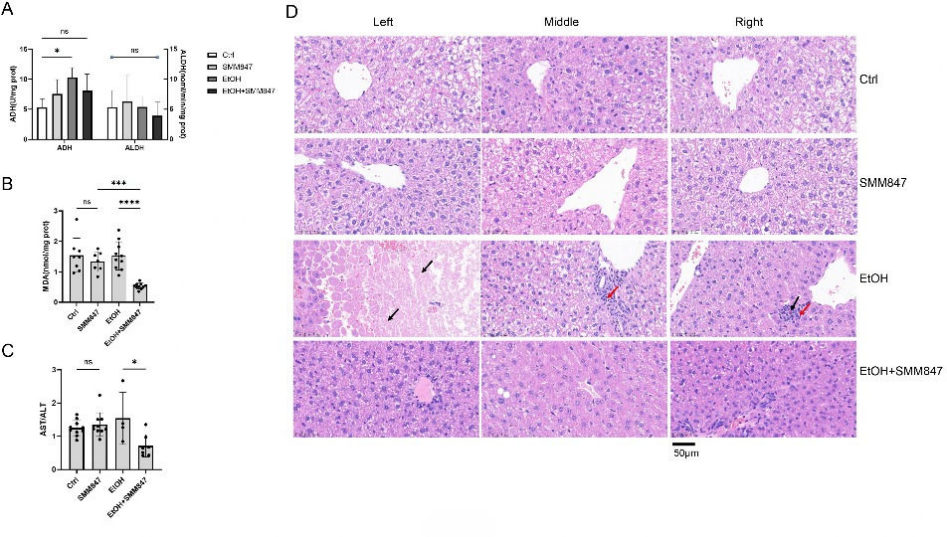
The effect of SMM847 on biochemical indexes in alcohol-modeled mice. (**A**) ADH and ALDH enzyme activities in the liver of mice. (**B**) The MDA content. (**C**) The AST/ALT ratio. (**D**) Hematoxylin and eosin (H&E) staining of the livers of alcohol-modeled mice. The red arrows point to the infiltrating inflammatory cells. The black arrows point to areas of necrosis in the liver cells. The left, middle, and right panels were selected randomly for H&E staining. Results are presented as mean values ± SD. *N* = 10, statistical significance was determined by Tukey’s multiple comparison test. *P < 0.05, **P < 0.01, ***P < 0.001, and ****P < 0.0001.

The measurement of key alcohol-metabolizing enzymes in the liver indicated that alcohol dehydrogenase (ADH) activity was significantly elevated in the EtOH group compared to the control group (Fig. 5A), suggesting an adaptive response to alcohol exposure. In contrast, the EtOH + SMM847 group showed no significant differences in ADH and ALDH activities compared to the control group. Based on these findings, we hypothesize that SMM847 may metabolize a substantial portion of alcohol in the intestine, using ethanol as a carbon source, thereby reducing systemic ethanol absorption and alleviating the metabolic burden on the liver. Furthermore, the EtOH group exhibited significantly higher levels of malondialdehyde (MDA) content (Fig. 5B) and an elevated AST/ALT ratio (Fig. 5C) compared to the EtOH + SMM847 group. These results suggest that under alcohol-induced stress, SMM847 alleviates alcohol-related impairments in nutrient absorption or metabolism by restoring hepatic metabolic function and enhancing endogenous antioxidant capacity, which may explain the body weight recovery during the mid-modeling phase.

Following euthanasia, liver tissues were collected from three randomly selected mice per group (*N* = 10 per group) for the hematoxylin and eosin (H&E) staining. Histopathological examination revealed extensive or focal hepatocyte necrosis with inflammatory cell infiltration in the portal areas of the EtOH group (Fig. 5D), confirming successful induction of alcohol-induced liver injury. In contrast, the other three groups showed no significant necrotic changes or inflammatory infiltration in hepatic tissues. These results demonstrate that SMM847 not only exhibits no adverse histopathological effects but also effectively attenuates alcohol-induced liver damage, indicating its potent hepatoprotective role.

## DISCUSSION

Gout (16) is an inflammatory condition triggered by the deposition of monosodium urate crystals in joint and periarticular tissues, which initiates an immune response and subsequent inflammation (17). Its predisposing factors can be categorized into genetic (18) and environmental factors (19). Hyperuricemia is a key risk factor for gout development, arising from either overproduction or impaired renal excretion of uric acid (20). In purine metabolism, purines are sequentially metabolized into hypoxanthine and xanthine, ultimately generating uric acid (21). The rationale underlying our experiment was to enable this microbe to utilize purines as a preferred carbon and energy sources in the intestinal lumen, thereby attenuating systemic uric acid burden. Experiments in a hyperuricemic rat model confirmed that SMM847 significantly lowers serum uric acid levels. However, it should be noted that purines contributing to uric acid production extend beyond inosine to guanosine, adenine, and others (22). No free purines were detected in the rat intestinal metabolome, which may be attributed to their efficient metabolic conversion by the gut microbiota. Relevant studies indicate that commensal intestinal microorganisms can rapidly degrade purines into end products such as pyruvate and short-chain fatty acids (23). At the same time, purine absorbed will be metabolized into uric acid. Compared with the model group, the experiment shows that the level of uric acid of the SMM847 group decreases, which demonstrates SMM847 has the ability to reduce the uric acid. Thus, the absence of purine signals in this experiment suggests their rapid microbial utilization and transformation within the intestinal environment, further supporting the important role of microbiota-mediated purine degradation in maintaining local purine homeostasis in the gut. Although this study demonstrates SMM847’s ability to degrade inosine efficiently *in vitro* and significantly reduce serum uric acid *in vivo*, the precise mechanistic basis—as well as the functional genes involved—remains unclear and warrants further investigation.

Besides purine metabolism, alcohol consumption is another major risk factor for gout (24). Alcohol intake accelerates purine catabolism, enhances uric acid production, and impairs renal uric acid excretion (25). Moreover, ethanol metabolism generates acetate (26), which lowers systemic pH and facilitates uric acid crystallization, thereby aggravating urate deposition and gout attacks. Since alcohol is primarily metabolized by ADH and ALDH (26), we screened SMM847 for its ADH and ALDH activities, hypothesizing that the strain could metabolize alcohol in the intestinal lumen, thereby reducing its systemic absorption. In alcohol-challenged mouse models, SMM847 significantly lowered blood ethanol levels, alleviated intoxication symptoms, shortened recovery time, and mitigated alcohol-induced liver histopathology. Nevertheless, as alcohol is predominantly absorbed in the stomach and small intestine and considering the harsh gastric environment for probiotic survival, it remains unclear whether and how SMM847 influences gastric alcohol absorption.

Hepatic MDA content is a classical indicator of oxidative stress and membrane damage in hepatocytes (27). The AST/ALT ratio helps assess the type, severity, and potential etiology of liver injury (28). In this study, neither the AST/ALT ratio nor MDA levels in the SMM847-treated group differed significantly from the control group, indicating that SMM847 does not adversely affect liver function or redox balance under normal physiological conditions, supporting its biosafety. However, under alcohol-induced stress, SMM847 exhibited strong regulatory and protective effects, indicating a context-dependent hepatoprotective potential. Notably, an AST/ALT ratio <1 in the SMM847 group may suggest mild hepatic impact, suggesting the need for future dose optimization to minimize any potential adverse effects.

In summary, SMM847 demonstrates a dual-function capacity to lower uric acid levels in rats and degrade blood ethanol in mice, effectively alleviating key risk factors for gout and alcohol-induced liver injury. These results validate its therapeutic candidacy and underscore the considerable potential of SMM847 as a foundational probiotic for developing therapeutic agents targeting both gout prevention and alcohol detoxification.

## MATERIALS AND METHODS

### Screening of microbial strains with potential uric acid-reducing capacities

The liquid culture incubated at 37°C with shaking for 24–48 h was centrifuged at 4,000 rpm for 10 min at 4°C using a refrigerated centrifuge. The supernatant was discarded, and the bacterial pellet was collected. Subsequently, 750 μL of 500 mg/L inosine in phosphate buffer (pH 6.8) was added to resuspend the pellet. The mixture was incubated at 37°C with shaking at 200 rpm for 1 h. The reaction was immediately terminated by heating in a boiling water bath at 100°C for 5 min, followed by cooling to room temperature. The sample was then centrifuged at 10,000 × *g* for 2 min, and the supernatant was collected and filtered through a 0.22 μm microporous membrane. An aliquot of 10 μL of the filtrate was injected into the high-performance liquid chromatography (HPLC) system for analysis. The chromatographic conditions were as follows: flow rate of 0.8 mL/min, column temperature maintained at 30°C, total run time of 15 min, and detection wavelength set at 260 nm. The mobile phase consisted of methanol and 0.2% acetic acid aqueous solution in a volume ratio of 5:95. For accurate quantification, a standard curve was established using a series of inosine standards (100, 200, 300, 400, and 500 mg/L; Beijing Solarbio Science & Technology Co., Ltd.), which exhibited excellent linearity (*R*² > 0.999) across the entire concentration range, thereby ensuring reliable quantification of samples with an initial inosine concentration of 500 mg/L. The inosine degradation rate was calculated using the formula (*C*_0_ − *C*_*t*_)/*C*_0_ × 100%, where *C*_0_ and *C*_*t*_ represent the inosine concentrations at 0 h and time *t*, respectively.

### Measurement of alcohol metabolism enzyme activity

The bacterial cells from liquid cultures incubated at 37°C with shaking for 24–48 h were collected by centrifugation at 9,000 rpm for 1 min. The supernatant was discarded, and the bacterial pellet was retained. A lysozyme solution was prepared by mixing 21 μL of 50 mg/mL lysozyme with 329 μL of 1× TE buffer, and 100 μL of this mixture was added to the pellet. After thorough pipetting and mixing, the suspension was allowed to stand for 20 min, followed by centrifugation at 4,000 rpm for 3 min. The resulting supernatant was discarded, and the pellet was resuspended in 100 μL of RIPA lysis buffer (Beyotime Institute of Biotechnology, Shanghai, China). The mixture was kept on ice for 20 min and then centrifuged at 12,000 rpm for 10 min at 4°C. The supernatant was carefully collected and retained for subsequent enzymatic assays. The activities of alcohol dehydrogenase (ADH) and aldehyde dehydrogenase (ALDH) were determined using commercial assay kits (Hefei Leier Biotechnology Co., Ltd.) in accordance with the manufacturer’s instructions.

### *In vitro* alcohol degradation potential assessment

The cultures were prepared and partitioned into two groups: (i) a bacterium-free control with 10% ethanol (EtOH), and (ii) 10% ethanol inoculated with the bacterial strain SMM847 (EtOH + SMM847) at an initial OD_600_ of 0.5–0.8. After immediate equilibration by vortexing, the zero-time point (*T*_0_) was recorded. Both sets were hermetically sealed to ensure strict anaerobiosis and incubated at 37°C under continuous orbital agitation. Aseptic aliquots were withdrawn at *T*_0_, *T*_1_ (2 h), *T*_2_ (4 h), and *T*_3_ (6 h); ethanol concentrations were quantified with the enzymatic assay kit (Suzhou Grees Biotech Co., Ltd.) following the manufacturer’s specifications.

### Bacterial labeling

CY5.5-d-lysine was dissolved in 100% dimethyl sulfoxide to a concentration of 2.5 mg/mL, aliquoted, and stored protected from light at 4°C. SMM847 was cultured overnight at 37°C with shaking at 950 rpm, then subcultured at a 2% inoculum into fresh MRS medium. When the bacterial culture reached the logarithmic growth phase (after 1 h of incubation at 37°C and 950 rpm), 20 μL of the CY5.5-d-lysine solution was added to achieve a final concentration of 50 μg/mL. The mixture was then incubated at 37°C with shaking at 950 rpm for 10 h. Subsequently, the bacteria were washed three times with ice-cold phosphate-buffered saline (PBS) and finally resuspended in 200 μL of PBS for subsequent analysis.

### *In vivo* tracking of fluorescently labeled SMM847

*In vivo* near-infrared fluorescence imaging to track the colonization of SMM847 in mice was performed using a small animal *in vivo* imaging system (IVIS Spectrum, USA) and its associated software (Living Image 4.7.2). Six 7-week-old female ICR mice under specific pathogen-free (SPF) conditions were used. Three mice in the control group were orally administered 200 μL of PBS, while three mice in the experimental group received 200 μL of PBS containing CY5.5-d-lys-SMM847. Near-infrared fluorescence imaging was conducted at 30 min, 1.5 h, 3 h, and 6 h post-administration. The fluorescent signals in the intestinal regions were quantified using the analysis software.

### *In vivo* uric acid-reducing capacity assessment

Twenty-four male SD adult rats (aged 8–10 weeks, weighing 200–220 g) were randomly divided into three equal groups. They were housed in SPF-grade rat cages maintained at 20°C–25°C, 50% ± 5% humidity, and a 12 h/12 h light-dark cycle. After 1 week of acclimation, rats underwent a 2-week modeling period. The SMM847 group was fed a high-purine customized rat diet (Shanghai Yitong Biotechnology Co., Ltd.), administered potassium oxonate dissolved in sodium carboxymethylcellulose, and gavaged with 1 × 10⁹ CFU SMM847 bacterial suspension. The model group was fed a high-purine customized rat diet, injected with potassium oxonate dissolved in sodium carboxymethylcellulose, and gavaged with an equal volume of physiological saline. The control group was fed a standard diet (Shanghai Yitong Biotechnology Co., Ltd.), injected with an equal volume of sodium carboxymethylcellulose aqueous solution, and gavaged with an equal volume of physiological saline. Blood samples were collected via retro-orbital bleeding on day 7 and day 14 of the modeling period. Prior to collection, rats were fasted for 6 h with free access to water. After standing at room temperature for 30 min, the blood was centrifuged at 3,500 × *g* for 15 min at 4°C to obtain serum. Serum uric acid levels were assayed using a cobas 8000 automatic biochemical analyzer (Roche Diagnostics).

### Metabolomics analysis of rat intestinal contents

Metabolites were extracted using a methanol–acetonitrile mixed solvent system. Briefly, 200 µL of each sample was precisely transferred into a 1.5 mL centrifuge tube, followed by the addition of 800 µL of ice-cold extraction solvent (methanol:acetonitrile = 1:1, vol/vol) containing four internal standards, including L-2-chlorophenylalanine (0.02 mg/mL). After vortexing for 30 s, the mixture was subjected to ultrasonic extraction for 30 min at 5°C (40 kHz). The samples were then kept at −20°C for 30 min, centrifuged at 13,000 × *g* for 15 min at 4°C, and the supernatant was collected and dried under a gentle nitrogen stream. The dried residue was reconstituted in 120 µL of reconstitution solution (acetonitrile:water = 1:1, vol/vol), vortexed again for 30 s, and sonicated for 5 min at 5°C. After centrifugation (13,000 × *g*, 10 min, 4°C), the supernatant was transferred to an injection vial with an insert for LCMS analysis. In addition, a quality control (QC) sample was prepared by pooling 20 µL of supernatant from each individual sample to monitor the stability of the analytical process. Metabolomic profiling was performed on an AB SCIEX UHPLC-Triple TOF 6600 system. Chromatographic separation was achieved on an ACQUITY UPLC HSS T3 column (100 mm × 2.1 mm, 1.8 µm; Waters, Milford, USA) maintained at 45°C. The mobile phase consisted of (i) water-acetonitrile (95:5, vol/vol) with 0.1% formic acid and (ii) acetonitrile-isopropanol-water (47.5:47.5:5, vol/vol/vol) with 0.1% formic acid, delivered at a flow rate of 0.40 mL/min. The injection volume was 10 µL. Mass spectrometric detection was carried out using electrospray ionization with data acquired in both positive and negative ion modes. Throughout the analytical sequence, a QC sample was injected after every 5–15 experimental samples to evaluate instrument performance and data stability.

### Establishment of the alcohol-exposed mouse model

Forty 8–10-week-old male 57BL/6J mice weighing 20–22 g were housed in SPF-grade mouse rooms maintained at 20°C–25°C, 50% ± 5% humidity, and a 12 h/12 h light-dark cycle. Control Lieber-DeCarli diet (TP4030C) and ethanol Lieber-DeCarli diet (TP4030D) were purchased from Shanghai Yitong Biotechnology Co., Ltd., Shanghai, China (29). After 1 week of acclimatization, mice were randomly divided into four groups of control (Ctrl), SMM847 group (SMM847), alcohol group (EtOH), and alcohol + SMM847 group (EtOH + SMM847). During the feeding regimen, the Lieber-DeCarli liquid diet was administered from day 1 to day 5, constituting Phase T1; from day 6 to day 20, the model liquid diet was provided, defining Phase T2; and day 21 was designated as Phase T3. Tail vein blood samples were collected 0.5 and 1.5 h after the final gavage, placed into heparin sodium tubes, and stored at 4°C for subsequent analysis. Nine hours after the final gavage, mouse serum and liver tissues were collected for further experimental procedures.

### Phenotypic analysis of the alcohol-exposed mouse model

Body weight of the mice was recorded on days 10, 15, and 20. Following a single ethanol gavage, the time to onset of intoxication and the total duration of intoxication were observed and recorded to calculate alcohol tolerance time—defined as the period from the initiation of intoxication to the loss of the righting reflex. The time of intoxication and recovery was documented to determine the sobering time, which refers to the interval from the initial loss of the righting reflex to its reappearance. Intoxication was defined as the loss of the righting reflex, indicated by the mouse remaining supine for more than 30 s. The survival rate was calculated as the ratio of the number of surviving mice at the end of the experiment to the initial number of mice.

### *In vivo* assessment of blood ethanol reduction capacity in the alcohol-exposed mouse model

Blood samples were collected from the tail vein at 0.5 h and 1.5 h after the final gavage administration. The samples were stored in heparinized tubes, and blood ethanol concentrations were determined using the ethanol assay kit (Suzhou Grees Biotech Co., Ltd.).

### Biochemical analysis of liver tissue and serum in the alcohol- exposed mouse model

Liver tissues from mice were homogenized, and the activities of ADH and ALDH were measured using the assay kits (Shanghai Preferred Biotechnology Co., Ltd.). The hepatic MDA content was determined with an MDA assay kit (Nanjing Jiancheng Bioengineering Institute). Serum levels of alanine aminotransferase (ALT) and aspartate aminotransferase (AST) were assessed using corresponding biochemical assay kits (Nanjing Jiancheng Bioengineering Institute).

## References

[1] Mehmood A, Iftikhar A, Chen X. 2024. Food-derived bioactive peptides with anti-hyperuricemic activity: a comprehensive review. Food Chem 451:139444. doi:10.1016/j.foodchem.2024.139444 38678657

[2] Kaneko K, Tsuruga K, Takayanagi F, Fukuuchi T, Yamaoka N, Seki R, Fujimori S. 2024. Daily amount of purine in commonly recommended well-balanced diets in Japan and overseas. Nutrients 16:4066. doi:10.3390/nu16234066 39683460 PMC11643512

[3] Yin H, Liu N, Chen J. 2022. The role of the intestine in the development of hyperuricemia. Front Immunol 13:845684. doi:10.3389/fimmu.2022.845684 35281005 PMC8907525

[4] Terkeltaub R, Dodd D. 2025. The gut microbiome in hyperuricemia and gout. Arthritis Rheumatol 77:955–965. doi:10.1002/art.43118 39829115 PMC12276925

[5] Wang Z, Li Y, Liao W, Huang J, Liu Y, Li Z, Tang J. 2022. Gut microbiota remodeling: a promising therapeutic strategy to confront hyperuricemia and gout. Front Cell Infect Microbiol 12:935723. doi:10.3389/fcimb.2022.935723 36034697 PMC9399429

[6] Major TJ, Takei R, Matsuo H, Leask MP, Sumpter NA, Topless RK, Shirai Y, Wang W, Cadzow MJ, Phipps-Green AJ, et al.. 2024. A genome-wide association analysis reveals new pathogenic pathways in gout. Nat Genet 56:2392–2406. doi:10.1038/s41588-024-01921-5 39406924

[7] FitzGerald JD. 2025. Gout. Ann Intern Med 178:ITC33–ITC48. doi:10.7326/ANNALS-24-03951 40063960

[8] Faller J, Fox IH. 1982. Ethanol-induced hyperuricemia: evidence for increased urate production by activation of adenine nucleotide turnover. N Engl J Med 307:1598–1602. doi:10.1056/NEJM198212233072602 7144847

[9] Yamanaka H. 1996. Alcohol ingestion and hyperuricemia. Nihon Rinsho 54:3369–3373. 8976122

[10] Li S, Jiang W, Zheng C, Shao D, Liu Y, Huang S, Han J, Ding J, Tao Y, Li M. 2020. Oral delivery of bacteria: basic principles and biomedical applications. J Control Release 327:801–833. doi:10.1016/j.jconrel.2020.09.011 32926886

[11] Liu Y, Li L, Feng J, Wan B, Tu Q, Cai W, Jin F, Tang G, Rodrigues LR, Zhang X, Yin J, Zhang Y. 2024. Modulation of chronic obstructive pulmonary disease progression by antioxidant metabolites from Pediococcus pentosaceus: enhancing gut probiotics abundance and the tryptophan-melatonin pathway. Gut Microbes 16:2320283. doi:10.1080/19490976.2024.2320283 38444395 PMC10936690

[12] Wang L, Liu Q, Chen Y, Zheng X, Wang C, Qi Y, Dong Y, Xiao Y, Chen C, Chen T, Huang Q, Zhai Z, Long C, Yang H, Li J, Wang L, Zhang G, Liao P, Liu YX, Huang P, Huang J, Wang Q, Chu H, Yin J, Yin Y. 2022. Antioxidant potential of Pediococcus pentosaceus strains from the sow milk bacterial collection in weaned piglets. Microbiome 10:83. doi:10.1186/s40168-022-01278-z 35650642 PMC9158380

[13] Jiang XW, Li YT, Ye JZ, Lv LX, Yang LY, Bian XY, Wu WR, Wu JJ, Shi D, Wang Q, Fang DQ, Wang KC, Wang QQ, Lu YM, Xie JJ, Li LJ. 2020. New strain of Pediococcus pentosaceus alleviates ethanol-induced liver injury by modulating the gut microbiota and short-chain fatty acid metabolism. World J Gastroenterol 26:6224–6240. doi:10.3748/wjg.v26.i40.6224 33177795 PMC7596634

[14] Chen W, Tian T, Zhou J, Yang D, Liang M, He Y, Yang S, Aikepa D, Sun Y. 2025. Safety evaluation of human-derived uric acid degrading Lacticaseibacillus paracasei M2a and its impact on gut microbiota. Probiotics Antimicrob Proteins. doi:10.1007/s12602-025-10562-x PMC12999659 40316867

[15] Zhou S, Wen X, Lessing DJ, Chu W. 2025. Uric acid-degrading Lacticaseibacillus paracasei CPU202306 ameliorates hyperuricemia by regulating uric acid metabolism and intestinal microecology in mice. Probiotics Antimicrob Proteins. doi:10.1007/s12602-025-10532-3 40205164

[16] Badshah M, Nadeem I, Ahmed I, Naim T, Fanciullo J. 2024. Gout: a rapid review of presentation, diagnosis and management. S D Med 77:81–86. 38986162

[17] Dalbeth N, Gosling AL, Gaffo A, Abhishek A. 2021. Gout. Lancet 397:1843–1855. doi:10.1016/S0140-6736(21)00569-9 33798500

[18] Pascart T, Ducoulombier V, Jauffret C. 2024. Early-onset gout. Joint Bone Spine 91:105704. doi:10.1016/j.jbspin.2024.105704 38336273

[19] Li R, Yu K, Li C. 2018. Dietary factors and risk of gout and hyperuricemia: a meta-analysis and systematic review. Asia Pac J Clin Nutr 27:1344–1356. doi:10.6133/apjcn.201811_27(6).0022 30485934

[20] Eckenstaler R, Benndorf RA. 2021. The role of ABCG2 in the pathogenesis of primary hyperuricemia and gout-an update. Int J Mol Sci 22:6678. doi:10.3390/ijms22136678 34206432 PMC8268734

[21] Furuhashi M. 2020. New insights into purine metabolism in metabolic diseases: role of xanthine oxidoreductase activity. Am J Physiol Endocrinol Metab 319:E827–E834. doi:10.1152/ajpendo.00378.2020 32893671

[22] Escudero C, Bertoglia P, Muñoz F, Roberts JM. 2013. Uric acid and purine plasma levels as plausible markers for placental dysfunction in pre-eclampsia. Rev Med Chil 141:895–902. doi:10.4067/S0034-98872013000700009 24356738

[23] Liu Y, Zhou Z, Jarman JB, Chen H, Miranda-Velez M, Terkeltaub R, Dodd D. 2025. Gut bacteria degrade purines via the 2,8-dioxopurine pathway. Nat Microbiol 10:2291–2305. doi:10.1038/s41564-025-02079-4 40770490 PMC12666987

[24] Nieradko-Iwanicka B. 2022. The role of alcohol consumption in pathogenesis of gout. Crit Rev Food Sci Nutr 62:7129–7137. doi:10.1080/10408398.2021.1911928 33866874

[25] Alharbi S, Aldubayan MA, Alhowail AH, Almogbel YS, Emara AM. 2024. Co-abuse of amphetamine and alcohol harms kidney and liver. Sci Rep 14:23400. doi:10.1038/s41598-024-74459-5 39379507 PMC11461853

[26] Paquot N. 2019. The metabolism of alcohol. Rev Med Liege 74:265–267. doi:10.1159/000499730 31206264

[27] Tsikas D. 2017. Assessment of lipid peroxidation by measuring malondialdehyde (MDA) and relatives in biological samples: analytical and biological challenges. Anal Biochem 524:13–30. doi:10.1016/j.ab.2016.10.021 27789233

[28] Liu H, Li H, Deng G, Zheng X, Huang Y, Chen J, Meng Z, Gao Y, Qian Z, Liu F, Lu X, Shi Y, Shang J, Yan H, Zheng Y, Shen Z, Qiao L, Zhang W, Wang X. 2024. Association of AST/ALT ratio with 90-day outcomes in patients with acute exacerbation of chronic liver disease: a prospective multicenter cohort study in China. Front Med 11:1307901. doi:10.3389/fmed.2024.1307901 PMC10993385 38576715

[29] Sánchez V, Baumann A, Kromm F, Yergaliyev T, Brandt A, Scholda J, Kopp F, Camarinha-Silva A, Bergheim I. 2024. Oral supplementation of choline attenuates the development of alcohol-related liver disease (ALD). Mol Med 30:181. doi:10.1186/s10020-024-00950-4 39425011 PMC11488139

